# Dual inhibition of MEK and PI3Kβ/δ–a potential therapeutic strategy in PTEN-wild-type docetaxel-resistant metastatic prostate cancer

**DOI:** 10.3389/fphar.2024.1331648

**Published:** 2024-01-22

**Authors:** Vicenç Ruiz de Porras, Adrià Bernat-Peguera, Clara Alcon, Fernando Laguia, Maria Fernández-Saorin, Natalia Jiménez, Ana Senan-Salinas, Carme Solé-Blanch, Andrea Feu, Mercedes Marín-Aguilera, Juan Carlos Pardo, Maria Ochoa-de-Olza, Joan Montero, Begoña Mellado, Albert Font

**Affiliations:** ^1^ CARE Program, Germans Trias i Pujol Research Institute (IGTP), Badalona, Barcelona, Spain; ^2^ Catalan Institute of Oncology, Badalona Applied Research Group in Oncology (B·ARGO), Badalona, Barcelona, Spain; ^3^ GRET and Toxicology Unit, Department of Pharmacology, Toxicology and Therapeutic Chemistry, Faculty of Pharmacy and Food Sciences, University of Barcelona, Barcelona, Spain; ^4^ Department of Biomedical Sciences, Faculty of Medicine and Health Sciences, Universitat de Barcelona, Barcelona, Spain; ^5^ IrsiCaixa AIDS Research Institute, Hospital Germans Trias i Pujol, Badalona, Barcelona, Spain; ^6^ Translational Genomics and Targeted Therapeutics in Solid Tumors Lab, Fundació de Recerca Clínic Barcelona–Institut d’Investigacions Biomèdiques August Pi i Sunyer (FRCB-IDIBAPS), Barcelona, Spain; ^7^ Department of Pathology, Germans Trias i Pujol University Hospital, Badalona, Barcelona, Spain; ^8^ Medical Oncology Department, Catalan Institute of Oncology, Badalona, Barcelona, Spain; ^9^ Medical Oncology Department, Hospital Clinic de Barcelona, Barcelona, Spain

**Keywords:** AZD8186, selumetinib, metastatic castration-resistant prostate cancer, PTEN status, taxane resistance

## Abstract

**Background:** Docetaxel remains the standard treatment for metastatic castration-resistant prostate cancer (mCRPC). However, resistance frequently emerges as a result of hyperactivation of the PI3K/AKT and the MEK/ERK pathways. Therefore, the inhibition of these pathways presents a potential therapeutic approach. In this study, we evaluated the efficacy of simultaneous inhibition of the PI3K/AKT and MEK/ERK pathways in docetaxel-resistant mCRPC, both *in vitro* and *in vivo*.

**Methods:** Docetaxel-sensitive and docetaxel-resistant mCRPC cells were treated with selumetinib (MEK1/2 inhibitor), AZD8186 (PI3Kβ/δ inhibitor) and capivasertib (pan-AKT inhibitor) alone and in combination. Efficacy and toxicity of selumetinib+AZD8186 were tested in docetaxel-resistant xenograft mice. CRISPR-Cas9 generated a PTEN-knockdown docetaxel-resistant cell model. Changes in phosphorylation of AKT, ERK and downstream targets were analyzed by Western blot. Antiapoptotic adaptations after treatments were detected by dynamic BH3 profiling.

**Results:** PI3K/AKT and MEK/ERK pathways were hyperactivated in PTEN-wild-type (wt) docetaxel-resistant cells. Selumetinib+AZD8186 decreased cell proliferation and increased apoptosis in PTEN-wt docetaxel-resistant cells. This observation was further confirmed *in vivo*, where docetaxel-resistant xenograft mice treated with selumetinib+AZD8186 exhibited reduced tumor growth without additional toxicity.

**Conclusion:** Our findings on the activity of selumetinib+AZD8186 in PTEN-wt cells and in docetaxel-resistant xenograft mice provide an excellent rationale for a novel therapeutic strategy for PTEN-wt mCRPC patients resistant to docetaxel, in whom, unlike PTEN-loss patients, a clinical benefit of treatment with single-agent PI3K and AKT inhibitors has not been demonstrated. A phase I-II trial of this promising combination is warranted.

## 1 Introduction

Although recent years have seen progress in the treatment of metastatic prostate cancer, the majority of patients progress to incurable metastatic castration-resistant prostate cancer (mCRPC) (Davies et al., 2019; Yamada and Beltran, 2021), which has a poor prognosis, with a median overall survival of approximately 3 years (Sartor and de Bono, 2018). Docetaxel is the cornerstone of first-line treatment for mCRPC and metastatic castration-sensitive prostate cancer (mCSPC) (Petrylak et al., 2004; Tannock et al., 2004; Sweeney et al., 2015), but its efficacy is limited by the development of tumor resistance (Ruiz de Porras et al., 2021a). For patients with mCSPC and mCRPC who have progressed after docetaxel treatment and have previously received new androgen receptor signaling inhibitors (ARSIs) (Yamada and Beltran, 2021), the available therapeutic options, such as radium-223, cabazitaxel, or sequential ARSI, have demonstrated limited efficacy, resulting in modest improvements in survival (de Bono et al., 2010). Improved understanding of the molecular mechanisms involved in mCRPC progression has led to the identification of predictive biomarkers and the development of new therapies with promising results (Davies et al., 2019), including PARP inhibitors, PSMA radioligands and immune checkpoint inhibitors (Yamada and Beltran, 2021). Nonetheless, treatment of mCRPC remains an important therapeutic challenge.

Loss of function of phosphatase and tensin homolog (PTEN) due to deletion or mutations can activate the PI3K/AKT pathway (Jamaspishvili et al., 2018). Dysregulation of this pathway occurs in approximately half of mCRPC patients (Abida et al., 2019) and is associated with taxane resistance, advanced tumor stage, risk of recurrence and worse prognosis (Liu et al., 2015; Jamaspishvili et al., 2018; Liu et al., 2020). In a recent trial with mCRPC patients, the combination of an AKT inhibitor (ipatasertib) plus abiraterone improved radiographic progression-free survival (rPFS) over placebo-plus-abiraterone in patients with PTEN-loss tumors (Sweeney et al., 2021). While PI3Kα alterations are frequent, PI3Kβ mutations are rare in mCRPC (Abida et al., 2019), but loss of PTEN protein expression creates a dependency on the PI3Kβ isoform (Wee et al., 2008). AZD8186 is a potent selective PI3Kβ/δ inhibitor that has demonstrated activity in PTEN-deficient breast (Bergholz et al., 2023) and prostate tumors given alone or combined with docetaxel (Hancox et al., 2015).

Preclinical models of PTEN-loss mCRPC have found a reciprocal relationship between the PI3K/AKT and the AR pathways, such that inhibition of one activates the other (Tortorella et al., 2023). Nevertheless, clinical trials with PI3K inhibitors plus AR-targeted therapies have demonstrated only a limited efficacy in mCRPC (Cham et al., 2021; Choudhury et al., 2022).

The MEK/ERK pathway regulates AR signaling, cell proliferation, cell differentiation, and treatment resistance. Hyperactivation of this pathway can be caused by genetic alterations of its upstream components, including *RAS*. This pathway is frequently dysregulated in several cancers, including mCRPC (Abida et al., 2019), although *RAS* mutations are rare (Cho et al., 2006). Selumetinib is a second-generation, selective, potent, non-ATP-competitive, allosteric MEK1/2 inhibitor that has been tested in different types of tumors at preclinical and clinical levels (Hedayat et al., 2022).

The MEK/ERK and PI3K/AKT pathways are interconnected (Mendoza et al., 2011) and present extensive crosstalk between them. Inhibition of the PI3K/AKT pathway results in upregulation of the MEK/ERK cascade and *vice versa*, leading to drug resistance (Lee et al., 2008; Wee et al., 2009; Park et al., 2015). Indeed, combination therapy with PI3K/AKT and MEK inhibitors could be a promising strategy in several tumor types, including mCRPC (Kinkade et al., 2008; Ewald et al., 2014; Park et al., 2015; Toren et al., 2016; Marques et al., 2020).

Although preclinical models have shown that inhibition of PI3Kβ or AKT enhances docetaxel cytotoxicity in PTEN-loss prostate cancer (Davies et al., 2012; Hancox et al., 2015), to the best of our knowledge, no studies have evaluated the effect of the combined inhibition of the PI3K/AKT and MEK/ERK pathways in docetaxel-resistant mCRPC patients. In order to explore the therapeutic potential of the combined inhibition of the two pathways, we have carried out a preclinical study in docetaxel-resistant mCRPC, investigating the antitumor efficacy of selumetinib, AZD8186 and capivasertib (a pan-AKT inhibitor), given as monotherapy or in combination.

## 2 Materials and methods

### 2.1 Cell lines

Docetaxel-resistant DU145-DR and PC3-DR cell lines had previously been generated from the PTEN-wild-type (PTEN-wt) DU145 (RRID: CVCL_0105) and the PTEN-loss PC3 (RRID: CVCL_0035) human mCRPC cell lines, respectively (Marin-Aguilera et al., 2012). Docetaxel IC50 doses were previously determined (Ruiz de Porras et al., 2021b) and are shown in Supplementary Table S1. Briefly, the DU145 and DU145-DR cells were cultured in RPMI (Thermo Fisher Scientific) and the PC3 and PC3-DR cells were cultured in Ham’s F12K medium (Thermo Fisher Scientific), supplemented with 10% of heat-inactivated FCS (Reactiva) and 1% penicillin-streptomycin (Thermo Fisher Scientific). Cell lines were cultured at 37°C in an atmosphere of 5% CO_2_, periodically tested for *mycoplasma* contamination, and authenticated by short tandem repeat profiling. Growth and molecular characteristics of the cell lines are summarized in Supplementary Table S2.

### 2.2 Drugs

Selumetinib, AZD8186, and capivasertib (Astra Zeneca Spain), were prepared in dimethyl sulfoxide (DMSO) at 50 mM and stored at room temperature (RT) (selumetinib) or 4°C (AZD8186 and capivasertib) protected from light.

### 2.3 Cell viability assay

Cell viability was measured using an MTT assay (Roche Diagnostics), seeding 6,000 to 8,000 cells/well in 96-well plates and treating with different drug concentrations for 72 h as previously described (Ruiz de Porras et al., 2016; Ruiz de Porras et al., 2021b).

### 2.4 Colony assay

Colony-formation assays were performed as previously described (Ruiz de Porras et al., 2016; Ruiz de Porras et al., 2021b). 500 cells/well were seeded and treated for 72 h, cultured in complete media for 10 days, washed, fixed with a methanol/acetic acid (3:1) solution, stained with crystal violet (0.5%) for 10 min and quantified using ImageJ software.

### 2.5 Western blot assay

Western blot assays were performed as previously described (Ruiz de Porras et al., 2016; Ruiz de Porras et al., 2021b) with the primary and secondary antibodies shown in Supplementary Table S3 and scanned in an Odyssey Imaging System (LICOR Biosciences).

### 2.6 Apoptosis assay

Annexin V (Alexa Fluor^®^ 647 Annexin V, 640912, BioLegend) and DAPI (62248, Thermo Fisher) were used and analyzing on a flow cytometry Fortessa 4 HTS instrument (BD Biosciences) as previously described (Alcon et al., 2022). Viable cells are negative for Annexin-V and DAPI, and cell death is expressed as 100% viable cells.

### 2.7 Dynamic BH3 profiling (DBP) assay

Antiapoptotic adaptations after treatments were detected by dynamic BH3 profiling (DBP) assay as previously described (Alcon et al., 2022). Briefly, DU145-DR cells were seeded at a seeding density of 3 × 10^5^ cells/well and were incubated with the single agents, their combinations or DMSO (control) for 96 h at 37°C. Cells were then stained with the viability marker Zombie Violet (423113, BioLegend) for 10 min at RT, washed with PBS and resuspended in MEB (150 mM mannitol, 10 mM hepes-KOH pH 7.5, 150 mM KCl, 1 mM EGTA, 1 mM EDTA, 0.1% BSA, 5 mM succinate). Simultaneously, BH3 peptide solutions were prepared in MEB with 0.002% digitonin (D141, Sigma-Aldrich). We used specific peptides against the most important antiapoptotic proteins: BAD peptide, which binds to BCL-2, BCL-xL and BCL-w; HRK peptide, which specifically binds to BCL-xL; and MS1 peptide, which binds to MCL-1 protein The final concentration of each peptide solution was: 10 µM of BAD BH3 peptide, 100 µM of HRK BH3 peptide, 10 µM of MS1 BH3 peptide, 25 µM of alamethicin (BML-A 150-0005, Enzo Life Sciences) and DMSO (control). 25 μL of cell suspensions were incubated with 25 µL of each peptide solution in a 96-well plate (3795, Corning) for 1 h at RT, followed by fixation with formaldehyde and further staining with cytochrome c antibody (Alexa Fluor^®^ 647—6H2.B4, 612310, BioLegend). Individual DBP analyses were performed in triplicates for DMSO, alamethicin, BAD, HRK, and MS1 BH3 peptides. The different analyses were performed with a high-throughput spectral flow cytometry Cytek AURORA instrument (CytekBio). The difference in % cytochrome c released between treated vs. non-treated cells after exposure to the specific peptides represents the antiapoptotic adaptations acquired by DU145-DR cells after treatment.

### 2.8 PTEN-knockdown with CRISPR/Cas9

PTEN knockdown was performed with CRISPR/Cas9 in the DU145-DR cell line. pLentiCRISPR v2 with sgRNA’s were purchased from the library pool deposited by Genscript Biotech. The sgRNA target sequences were as follows: ACC​GCC​AAA​TTT​AAT​TGC​AG, TTA​TCC​AAA​CAT​TAT​TGC​TA and ACA​GAT​TGT​ATA​TCT​TGT​AA. Briefly, lentiviruses were produced in A293T transfected with pLentiCRISPR v2, a packaging and envelope plasmid using a lipofectamine 2000 protocol according to the manufacturer’s instructions. DU145-DR cells were infected with the lentivirus at 60% confluence in serum and antibiotic-free Opti-MEM. After 24 h, puromycin was added at 2 μg/mL with fresh medium as a selection marker. Clonal selection was performed by seeding 0.5 cell/well in a 96-well plate. The selected clone was expanded and was used for the subsequent experiments.

### 2.9 Preclinical assays *in vivo*

Animal housing, handling, and all experimental procedures involving mice (reference No. 11501) were ethically approved by the Institutional Review Board at the Centre for Comparative Medicine and Bioimage (CMCiB)–Germans Trias i Pujol Research Institute (Badalona, Barcelona, Spain) and were authorized by Spanish authorities for implementation at the CMCiB which is accredited by AAALAC International. Protocols adhered to the guidelines of European Directive 2010/63/UE, the Federation of European Laboratory Animal Science Associations, the Animal Welfare Act, and “The Guide for the Use and Care of Laboratory Animals’.

First, we evaluated the toxicity of the AZD8186 + selumetinib combinations. For this purpose, two BALB/c (nu/nu) mice per group were treated during 3 weeks as following: 1) AZD8186 40 mg/kg + selumetinib 10 mg/kg; 2) AZD8186 20 mg/kg + selumetinib 5 mg/kg.

Next, to determine the effect of pharmacologic inhibition of PI3K and MEK, as monotherapy or in combination, 8 × 10^6^ DU154-DR cells were injected subcutaneously (resuspended in 100 mL of RPMI and mixed with 100 mL of Matrigel basement membrane) in each back-skin flank of BALB/c (nu/nu) mice. When tumors were detectable (approximate volume of 180 mm^3^), mice were randomly assigned to a control or different treatment groups (n = 6 mice/group), as follows: 1) vehicle (control; HPMC 0.5% + 0.1% Tween80 in sterile water); 2) AZD8186 (40 mg/kg by oral gavage twice daily; diluted in HPMC 0.5% + 0.1% Tween80 sterile water); 3) selumetinib (10 mg/kg by oral gavage twice daily; diluted in HPMC 0.5% + 0.1% Tween80 in sterile water); 4) selumetinib+AZD8186 (same doses and regimen used in monotherapy) and 5) cabazitaxel (5 mg/kg by intraperitoneal injection once a week; diluted in 10% DMSO + 90% corn oil) which was used as the control treatment arm, as it is the standard treatment for patients with docetaxel-resistant mCRPC. Tumor growth was monitored by measuring tumor diameters with a caliper every 2 days. Tumor volume was calculated using the formula V = ½ (Length × Width^2), where Length represents the long diameter and Width corresponds to the short diameter. Mouse weight (in grams) was measured using a precision balance every 2 days. When tumors in the vehicle and monotherapy arms reached a critical size, all mice were euthanized, and tumor samples were collected. The tumors were then collected as fresh samples to check the inhibition of the signaling pathways and proliferation markers by Western blot.

### 2.10 Statistical analysis

In all functional assays *in vitro*, data are presented as mean ± SEM of at least 3 independent biological replicates with three internal technical replicates and the statistical analysis was performed with Graphpad Prism V.4 software. Statistical differences in cell viability were determined by graphic representation of dose-response curves and subsequent non-linear regression analysis and F-test (Figure 3G). For viability (bar graphs), colony formation, and apoptosis assays, *p*-values were calculated using a two-tailed Student’s t-test been the null hypothesis that no differences exist between the two groups compared in each experiment, experimental and control and values ≤ 0.05 were considered significant. Antiapoptotic adaptations after treatments were calculated using a two-way ANOVA test for multiple comparisons. One-way analysis of variance (ANOVA) was used to calculate the significance of the difference between the vehicle and each treatment group in the *in vivo* analysis. Calcusyn software (Biosoft, Cambridge, UK) was used to calculate the combination index (CI), where CI < 1 indicates synergistic and CI > 1 antagonistic interactions. All statistical analyses were performed with GraphPad Prism software. Significance was set at *p* ≤0.05.

## 3 Results

### 3.1 PI3K/AKT and MEK/ERK pathways are hyperactivated in docetaxel-resistant mCRPC cells

**FIGURE 1:**
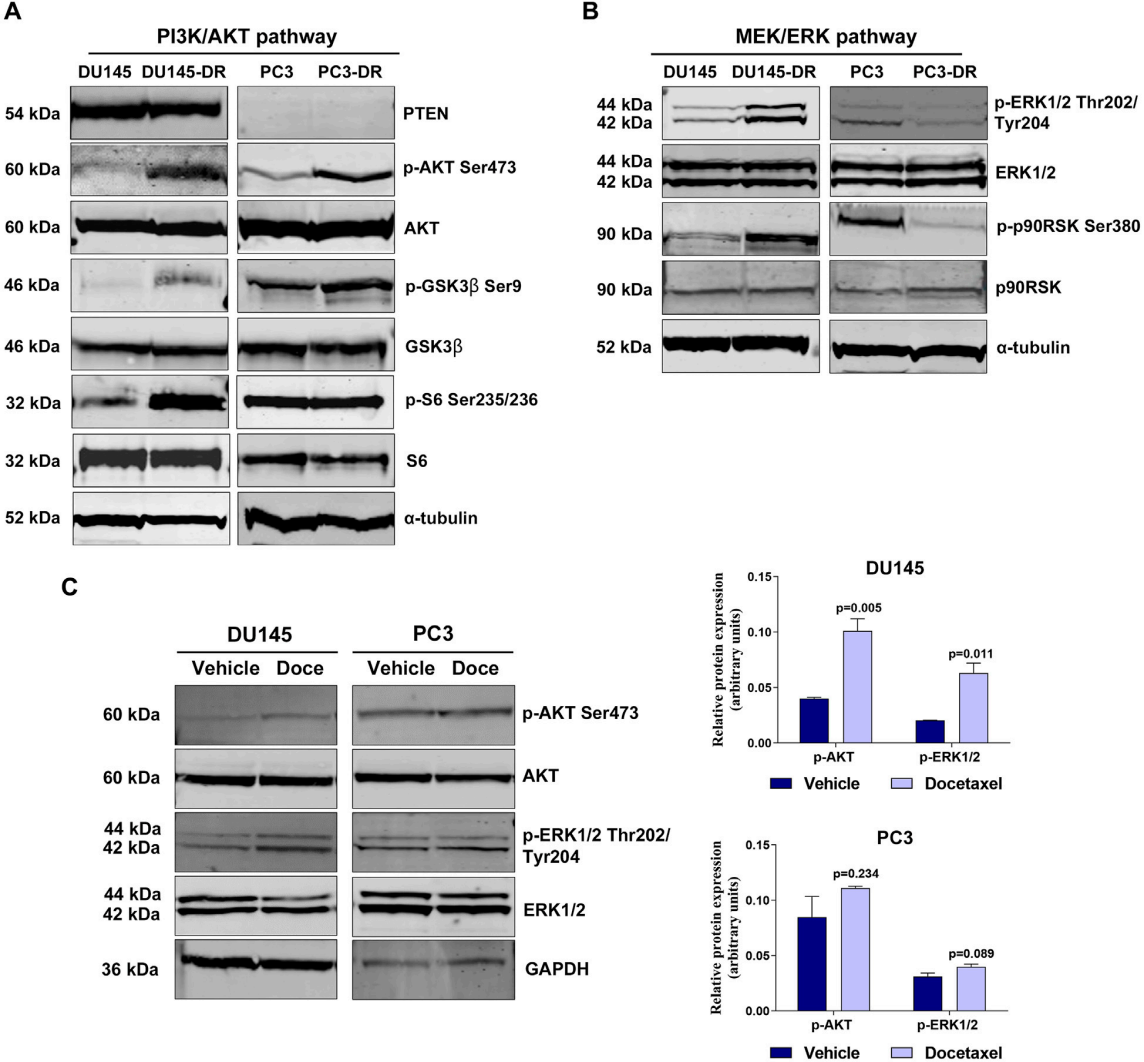
PI3K/AKT and MEK/ERK pathways are hyperactivated in docetaxel-resistant metastatic castration-resistant prostate cancer cell lines. **(A)** Representative Western blot images showing basal protein expression levels in the DU145, DU145-DR, PC3 and PC3-DR cell lines: phosphorylated AKT (p-AKT Ser473), phosphorylated GSK3β (p-GSK3β Ser9), phosphorylated S6 (p-S6 Ser235/236), PTEN, AKT, GSK3β and S6. α-tubulin was used as endogenous control. **(B)** Representative Western blot images showing basal protein expression levels of phosphorylated ERK1/2 (p-ERK1/2 Thr202/Tyr204), phosphorylated p90RSK (p-p90RSK Ser380), ERK1/2 and p90RSK in the DU145, DU145-DR, PC3 and PC3-DR cell lines. α-tubulin was used as endogenous control. **(C)** Western blot analysis (left) and bar graphs (right) showing protein expression changes of phosphorylated AKT (p-AKT) and phosphorylated ERK (p-ERK1/2) in DU145 and PC3 cells after treatment with docetaxel (Doce) at 6 and 12 nM, respectively, for 72 h α-tubulin was used as endogenous control. Results shown were obtained from at least three independent biological replicates. *p*-values were calculated using a two-tailed Student’s t-test. *p*-value relative to vehicle.

We determined the activation status of the PI3K/AKT and MEK/ERK pathways in the parental DU145 and PC3 cells and in their corresponding docetaxel-resistant derived cell models, DU145-DR and PC3-DR. As expected (Lee et al., 2008), the PTEN-loss PC3 cells had higher levels of phosphorylated AKT than the PTEN-wt DU145 cells, which had minimal endogenous AKT phosphorylation (Figure 1A). AKT was hyperphosphorylated at Ser473 in DU145-DR and PC3-DR cells compared to its parental docetaxel-sensitive cell lines DU145 and PC3, respectively (Figure 1A). Levels of phosphorylated S6 ribosomal protein at Ser235/236 were higher in DU145-DR as compared to DU145 (Figure 1A). Additionally, phosphorylation levels of glycogen synthase kinase‐3β (GSK3β) at Ser9 were elevated in both docetaxel-resistant cell lines compared to their respective parental cell lines (Figure 1A), since AKT phosphorylates multiple downstream oncogenic proteins, including S6 (Sekulic et al., 2000) and GSK-3β (Fang et al., 2000).

When looking into MEK/ERK pathway in the same cell lines, we observed relevant differences in pathway activation. PTEN-wt docetaxel-resistant cells had increased pathway activation than docetaxel sensitive cell lines. Indeed, ERK1/2 phosphorylation levels at Thr202/Tyr204 were higher in DU145-DR than in DU145. Conversely, PTEN-loss cells lines presented increased pathway activation in docetaxel-sensitive cells, as indicated by higher ERK1/2 phosphorylation levels at Thr202/Tyr204 in PC3 than in PC3-DR cells (Figure 1B). In line with these findings, phosphorylated p90RSK exhibited similar behavior as phospho-ERK1/2 across the different cell lines (Figure 1B), probably due to the fact that activated ERK1/2 phosphorylates cytoplasmic signaling proteins, including p90RSK (McCubrey et al., 2007). Furthermore, 72 h-docetaxel treatment promoted a significant increase in AKT (*p* = 0.005) and ERK (*p* = 0.019) phosphorylation in DU145 cells (Figure 1C), suggesting a potential adaptive response of these cells to docetaxel exposure, which might contribute to the development of drug resistance.

These results suggest that docetaxel resistance is associated with increased activation of the PI3K/AKT pathway in a PTEN independent manner, and can be accompanied with increased activation of the MEK/ERK pathway in PTEN-wt cells. Additionally, docetaxel treatment showed to activate these pathways *in vitro*. Therefore, combining PI3K/AKT and MEK/ERK inhibitors could be an attractive therapeutic approach to increase tumor cell death in docetaxel-resistant mCRPC.

### 3.2 Combination treatment with selumetinib+AZD8186 synergistically decreased cell viability and induced apoptosis in PTEN-wt docetaxel-resistant mCRPC cells

**FIGURE 2:**
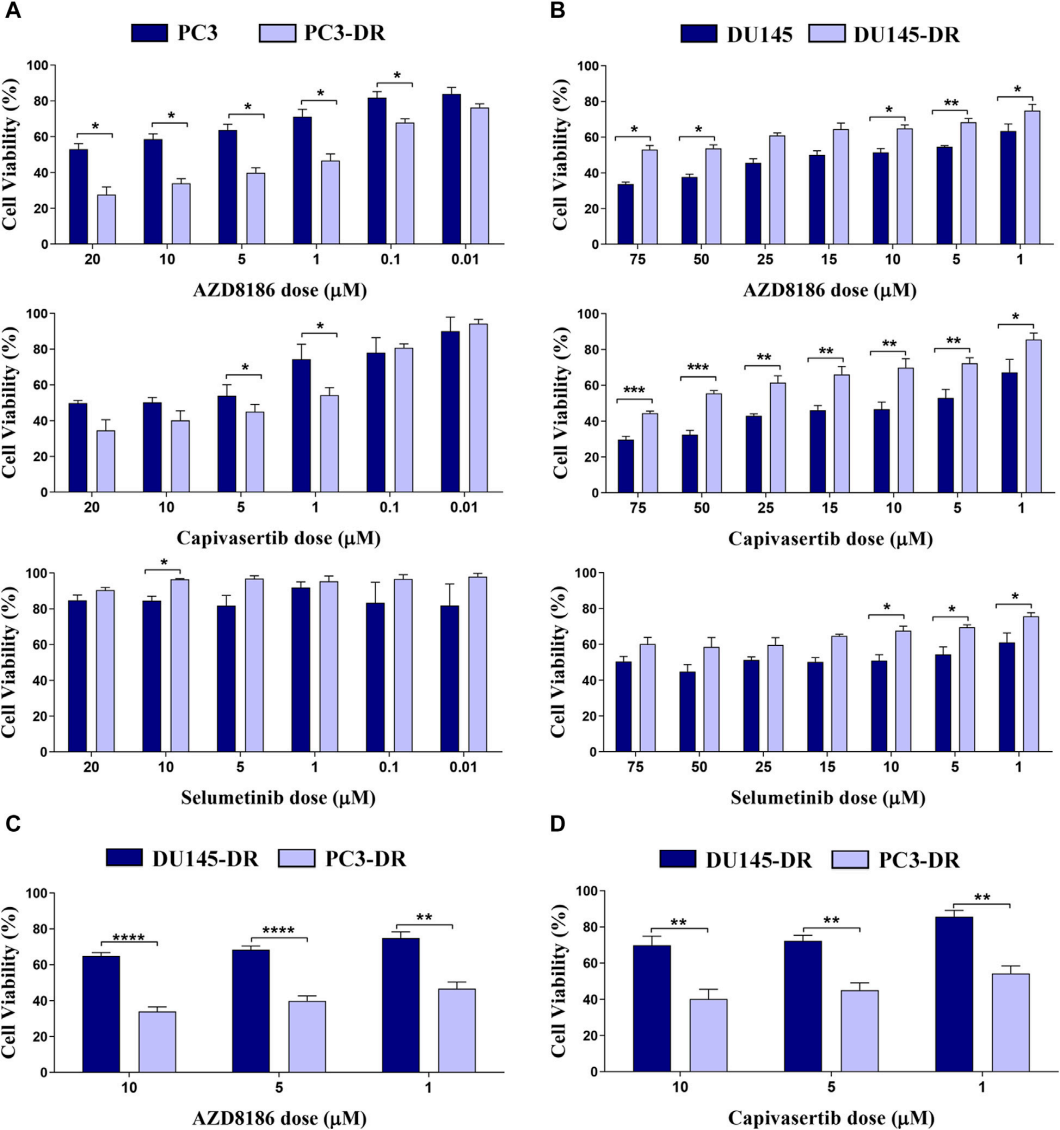
PI3K and AKT inhibitors promote a significant decrease in cell viability in PTEN-loss PC3-DR but not in PTEN-wild-type DU145-DR cells. **(A)** Bar graphs representing mean ± SEM percentage of cell viability after 72-h treatment with AZD8186 (upper panel), capivasertib (middle panel) or selumetinib (lower panel) at the indicated doses in PC3 and PC3-DR cells. **(B)** Bar graphs representing mean ± SEM percentage of cell viability after 72-h treatment with AZD8186 (upper panel), capivasertib (middle panel) or selumetinib (lower panel) at the indicated doses in DU145 and DU145-DR cells. Results shown were obtained from at least three independent biological replicates. *p*-values were calculated using a two-tailed Student’s t-test. *p ≤0.05; **p ≤0.01, ***p ≤0.001 relative to viability of parental cell lines **(C)** Bar graphs representing mean ± SEM percentage of cell viability after 72-h treatment with AZD8186 at the indicated doses in DU145-DR and PC3-DR cells. **(D)** Bar graphs representing mean ± SEM percentage of cell viability after 72-h treatment with capivasertib at the indicated doses in DU145-DR and PC3-DR cells. *p*-values were calculated using a two-tailed Student’s t-test. **p ≤ 0.01, ***p ≤0.001, **** *p* ≤ 0.0001 relative to viability of DU145-DR cells. Results shown were obtained from at least three independent biological replicates.

We first explored the individual effects of selumetinib (a MEK1/2 inhibitor), AZD8186 (a PI3Kβ/δ inhibitor), and capivasertib (a pan-AKT inhibitor) in all four cell lines. As expected, PTEN deficient cells showed strong sensitivity to both AZD8186 and capivasertib as is demonstrated by decreased cell viability in both PC3 and PC3-DR cell lines (Figure 2A). Moreover, cell viability was more reduced by PI3Kβ/δ (Figure 2A; upper panel) and AKT (Figure 2A; middle panel) inhibitors in docetaxel-resistant PTEN-loss PC3-DR cell lines when compared to docetaxel-sensitive PC3 cells, whilst MEK inhibitor selumetinib showed no effect on cell viability (Figure 2A; lower panel). In contrast, PTEN-wt DU145 cells were more sensitive to AZD8186 (Figure 2B; upper panel) and to capivasertib (Figure 2B; middle panel) than docetaxel-resistant DU145-DR cells. Importantly, PC3-DR cells were significantly more sensitive to AZD8186 and capivasertib than DU145-DR (Figures 2C, D). Overall, these results suggest that both PTEN status and docetaxel resistance modulate pathway inhibition sensitivity in our cell models.

**FIGURE 3:**
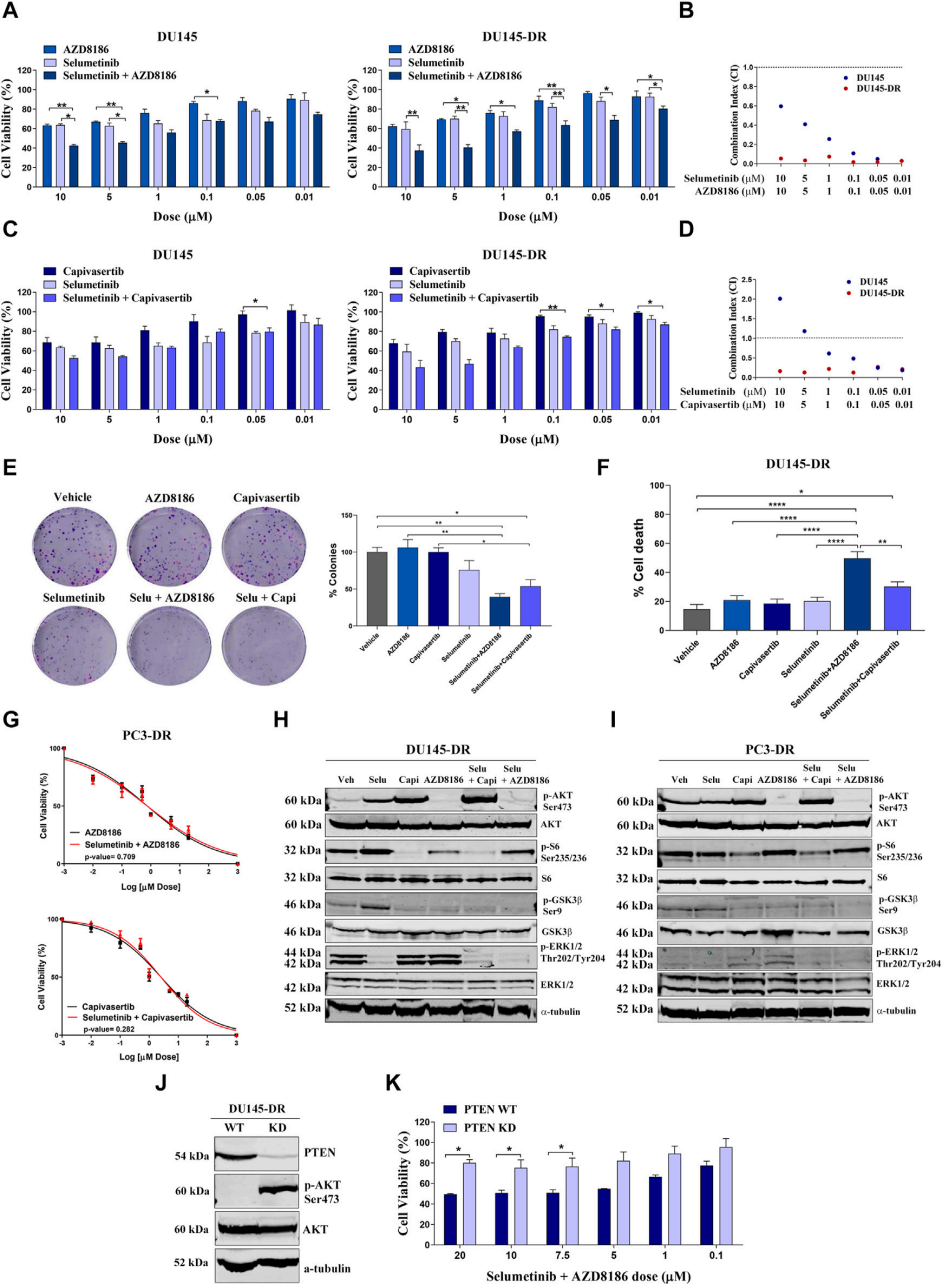
Selumetinib+AZD8186 synergistically decrease cell viability and induces apoptosis in PTEN-wild-type (PTEN-wt) docetaxel-resistant metastatic castration-resistant prostate cancer cell lines. **(A)** Bar graphs representing mean ± SEM percentage of cell viability after 72-h treatment with AZD8186, selumetinib or selumetinib+AZD8186 at the indicated doses in the PTEN-wt DU145 and DU145-DR cells. *p*-values are relative to the indicated treatment. **(B)** Dot plot representing combination index (CI) values calculated for selumetinib+AZD8186 at the indicated doses in DU145 (blue dots) and DU145-DR (red dots) cells. **(C)** Bar graphs representing mean ± SEM percentage of cell viability after 72-h treatment with capivasertib, selumetinib or selumetinib + capivasertib at the indicated doses in the PTEN-wt DU145 and DU145-DR cells. *p*-values are relative to the indicated treatment. **(D)** Dot plot representing CI values calculated for selumetinib + capivasertib at the indicated doses in DU145 (blue dots) and DU145-DR (red dots) cells. **(E)** Representative colony formation images (left panel) and bar graph representing the percentage (mean ± SEM) of colonies (right panel) in DU145-DR cells after 72 h of the indicated treatments. *p*-values are relative to vehicle or the indicated treatment. **(F)** Bar graph representing the percentage (mean ± SEM) of late apoptotic cells after 120 h of 5 µM of the indicated treatments. *p*-values are relative to vehicle or the indicated treatment. **(G)** Dose response curves for PC3-DR cells after treatment with selumetinib + AZD8186 (upper panel) or selumetinib + capivasertib (lower panel) at 0-20 µM for 72 h (mean ± SEM). **(H)** Representative Western blot images showing protein expression changes of the indicated proteins in DU145-DR cells after 72 h of 5 µM of the indicated treatments. α-tubulin was used as endogenous control. **(I)** Representative Western blot images showing protein expression changes of the indicated proteins in PC3-DR cells after 72 h of 5 µM of the indicated treatments. α-tubulin was used as endogenous control. **(J)** Representative Western blot images showing protein expression changes of PTEN and p-AKT in DU145-DR cells after PTEN knockdown (KD) by CRISPR Cas9. α-tubulin was used as endogenous control. **(K)** Bar graph representing the percentage (mean ± SEM) of cell viability after 72-h treatment with selumetinib + AZD8186 at the indicated doses in PTEN-wt DU145-DR and PTEN KD DU145-DR cells. *p*-values are relative to PTEN-wt DU145-DR cells. All results shown were obtained from at least three independent biological replicates. *p*-values were calculated using a two-tailed Student’s t-test. *p ≤ 0.05; **p ≤ 0.01; ****p ≤0.0001.

We moved forward to characterize combination treatments in our cell lines. We observed that in PTEN-wt cells, the combination of selumetinib + AZD8186 and selumetinib + capivasertib induced higher cell death than either drug alone (Figures 3A–D). This decrease in proliferation was accompanied by decreased colony formation (Figure 3E) and increased apoptosis (Figure 3F) in a highly synergistic manner in both PTEN-wt DU145 and DU145-DR cells, with the greatest cytotoxicity observed with selumetinib+AZD8186 in the DU145-DR cells. In contrast, the addition of selumetinib did not enhance the effectiveness of either capivasertib (*p* = 0.282) or AZD8186 (*p* = 0.709) in PC3-DR cells (Figure 3G), suggesting that double pathway inhibition may be especially relevant in docetaxel-resistant PTEN-wt cells.

In order to better understand these results, we explored possible post-treatment antiapoptotic adaptations which could be preventing cytotoxicity. We observed more cytochrome c release after treatment with selumetinib+capivasertib (*p* = 0.021) than after selumetinib+AZD8186 (*p* = 0.503) with the MS1 peptide, indicating an antiapoptotic adaptation through MCL-1 induced by the combination of selumetinib+capivasertib. This could partly explain the differences in apoptosis induction between the two treatments since the lower effectiveness of selumetinib+capivasertib could be due to the adaptation of cancer cells to therapy through the MCL-1 antiapoptotic protein (Supplementary Figure S1).

To decipher the effect of pathway inhibition on our cell lines, we then used Western blot to assess the activity of selumetinib, AZD8186, capivasertib, selumetinib+AZD8186 and selumetinib+capivasertib in DU145-DR (Figure 3H) and PC3-DR (Figure 3I) cells. In DU145-DR cells, treatment with capivasertib alone reduced phosphorylation of S6 and GSK3β but increased AKT phosphorylation, likely due to the stabilization of AKT in an inactive hyperphosphorylated form (Okuzumi et al., 2009). Treatment with AZD8186 alone strongly suppressed AKT and GSK3β phosphorylation and–to a lesser degree–S6 phosphorylation. Treatment with selumetinib as a single agent decreased ERK1/2 phosphorylation and increased AKT, S6 and GSK3β phosphorylation (Figure 3H). In PC3-DR cells, both capivasertib and AZD8186 increased ERK1/2 phosphorylation (Figure 3I). These pathway inhibition effects show activation of compensatory pathways when inhibiting a single signaling cascade.

Regarding combination treatments, selumetinib+AZD8186 and selumetinib+capivasertib both decreased activation of AKT, ERK and their downstream targets, with the exception of S6 after selumetinib+AZD8186 exposure in DU145-DR and PC3-DR cells (Figures 3H, I), effectively showing inhibition of both pathways and avoiding compensatory mechanisms.

Finally, to examine the role of PTEN status on the synergistic effect of selumetinib+AZD8186 in docetaxel-resistant cells, we knocked down PTEN in DU145-DR cells obtaining an 85%-90% reduction in protein expression. These PTEN-knockdown DU145-DR cells showed a large increase in AKT phosphorylation (Figure 3J) and were more resistant to selumetinib+AZD8186 than the PTEN-wt DU145-DR cells (Figure 3K).

These findings suggest that PTEN-wt docetaxel-resistant mCRPC cells are sensitive to selumetinib+AZD8186 and that this sensitivity is in part mediated by PTEN status.

### 3.3 Selumetinib+AZD8186 reduces tumor growth in PTEN-wt docetaxel-resistant xenograft mouse models

To test the effectiveness and toxicity of selumetinib+AZD8186 *in vivo*, we examined the effect of this combination in a DU145-DR-derived xenograft mouse model.

**FIGURE 4:**
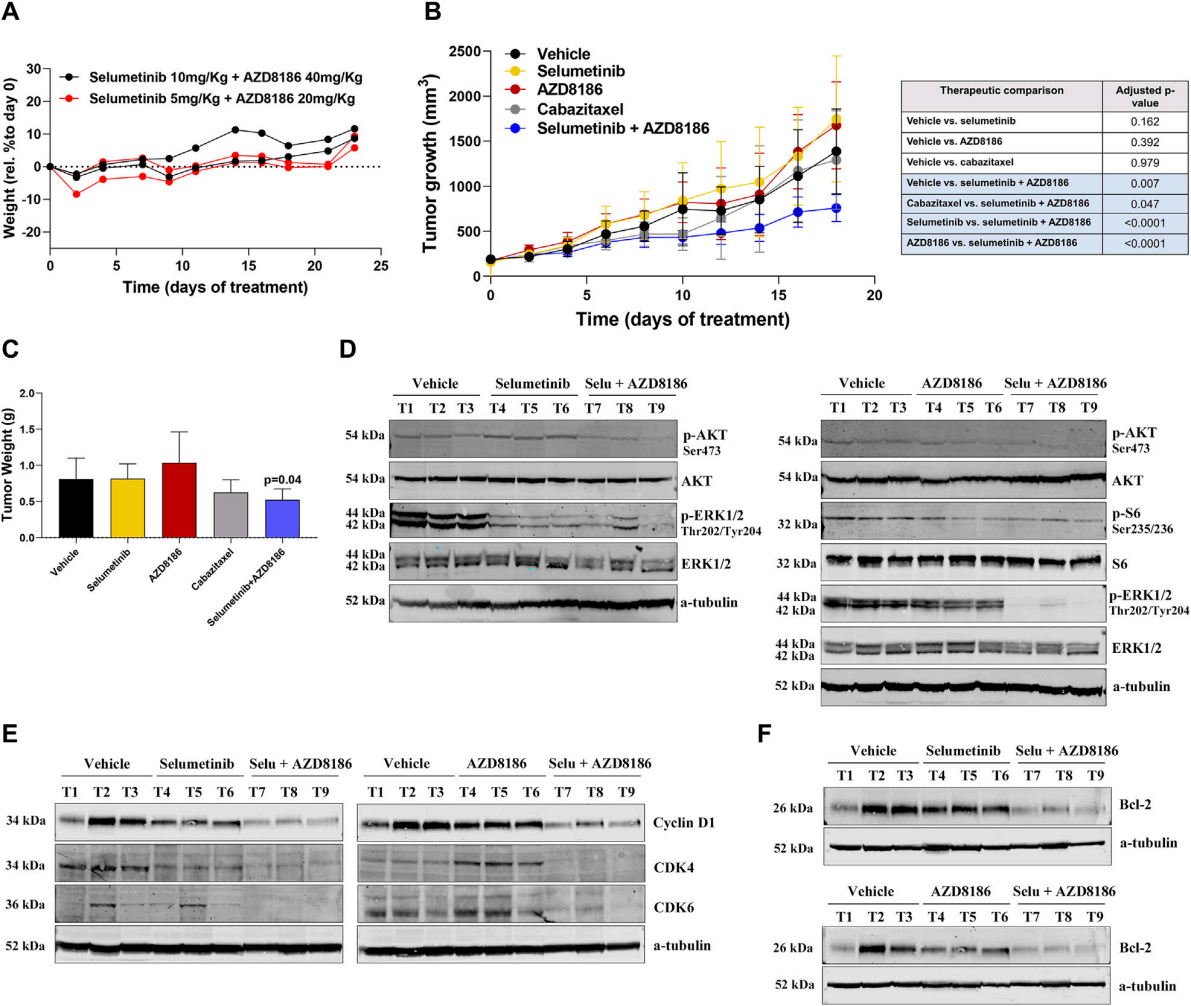
Selumetinib+AZD8186 reduces tumor growth in PTEN-wild-type docetaxel-resistant xenograft mouse models. **(A)** Body weight variation curves (percentage compared to initial weight) for BALB/c (nu/nu) mice treated (*n* = 2 mice/group) with the indicated drugs for 23 days. **(B)** Tumor growth curves for DU145-DR-derived xenograft models (*n* = 6 mice/group) treated with the indicated treatments or vehicle for 18 days. Differences in tumor growth were assessed with ANOVA. **(C)** Bar graph representing the percentage (mean ± SEM) of tumor weight after the indicated treatments for 18 days. The *p*-value is relative to vehicle. **(D)** Representative Western blot images showing p-AKT, p-ERK1/2 and p-S6 protein expression changes in tumor tissues from DU145-DR-derived xenograft mice exposed to the indicated treatment or vehicle. α -tubulin was used as an endogenous control. **(E)** Representative Western blot images showing Cyclin D1, CDK4 and CDK6 protein expression changes in tumor tissues from DU145-DR-derived xenograft mice exposed to the indicated treatment or vehicle **(F)** Representative Western blot images showing Bcl-2 protein expression changes in tumor tissues from DU145-DR-derived xenograft mice exposed to the indicated treatment or vehicle. α-tubulin was used as an endogenous control.

We first evaluated the toxicity of selumetinib+AZD8186 in BALB/c (nu/nu) mice. Importantly, the combination of selumetinib+AZD8186 showed no toxicity in the animals at any of the tested doses (Figure 4A). Regarding treatment efficacy, a significant reduction in tumor growth was observed after treatment with selumetinib+AZD8186 as compared to vehicle (45% tumor reduction; *p* = 0.0040), cabazitaxel (35% tumor reduction; *p* = 0.047), selumetinib (52% tumor reduction; *p* <0.0001) or AZD8186 (50% tumor reduction; *p* <0.0001) when administered as monotherapy (Figure 4B). Accordingly, we also observed a significant decrease in tumor weight after treatment with selumetinib +AZD8186 as compared to vehicle (*p* = 0.04) (Figure 4C). In contrast, no significant reduction in tumor growth was found in mice treated with selumetinib, AZD8186 or cabazitaxel alone (Figure 4B).

In line with our *in vitro* results, Western blot showed that AZD8186 decreased AKT and S6 phosphorylation, while selumetinib decreased ERK phosphorylation but increased AKT phosphorylation. As expected, the combined treatment resulted in decreased phosphorylation of both AKT and ERK (Figure 4D). Analysis of tumor specimens confirmed that the antitumor activity of selumetinib+AZD8186 was associated with a significant reduction in protein expression of the Cyclin D1/CDK4/CDK6 proliferation complex, thereby inducing cell cycle arrest at the G1/S checkpoint (Figure 4E) as well as with a decrease in Bcl-2 antiapoptotic protein expression (Figure 4F).

## 4 Discussion

Taxane resistance is a major obstacle to treatment efficacy in mCRPC patients. It is well-recognized that hyperactivation of the PI3K/AKT pathway–often due to PTEN loss–and of the MEK/ERK pathway play a key role in this resistance (Liu et al., 2015; Liu et al., 2020). In the present preclinical study, we have evaluated the dual inhibition of the PI3K/AKT and MEK/ERK pathways *in vitro* and *in vivo* and found it to be effective in PTEN-wt cells and xenograft mouse models.

In line with previous findings (Lee et al., 2008), we observed that docetaxel resistance was associated with hyperactivation of the MEK/ERK pathway in PTEN-wt DU145 cells but not in PTEN-loss PC3 cells, suggesting that AKT hyperactivation may well be related to MEK/ERK inactivation in PTEN-loss cells. However, the PI3K/AKT pathway was hyperactivated in both PTEN-wt DU145-DR and PTEN-loss PC3-DR cells, corroborating the well-known role of this pathway in taxane resistance (Liu et al., 2015; Liu et al., 2020).

PI3K, AKT and MEK inhibition using specific small kinase inhibitors has been proposed as an effective therapy in several tumor types, including mCRPC (Davies et al., 2012; Hancox et al., 2015; Park et al., 2015). Preclinically, several studies have demonstrated that both AKT and PI3K inhibitors have single-agent activity across prostate cancer cell lines and tumor xenograft models with PTEN loss (Hancox et al., 2015; Marques et al., 2015)**.** However, results in the clinical setting have so far been inconclusive. For instance, the IPATential150 trial showed that ipatasertib-plus-abiraterone slightly improved rPFS compared to placebo-plus-abiraterone among patients with mCRPC and PTEN-loss tumors (Sweeney et al., 2021) but not in those with PTEN-wt tumors. Several preclinical studies (Davies et al., 2012; Hancox et al., 2015) have reported that pharmacological inhibition of PI3Kβ or AKT enhances docetaxel cytotoxicity in mCRPC. In this context, the PROcaid randomize phase II trial carried out in mCRPC patients showed that docetaxel-plus-capivasertib did not improve rPFS compared with docetaxel alone (Crabb et al., 2022) but surprisingly conferred a benefit in survival, mainly in patients previously treated with AR inhibitors. The ongoing CAPItello-280 phase III trial (ClinicalTrials.gov NCT05348577) is evaluating the efficacy of docetaxel-plus-capivasertib vs. docetaxel alone in mCRPC patients who have progressed to a previous treatment with ARSIs.

In line with the IPATential150 study results (Sweeney et al., 2021), we have found that the PTEN-loss PC3 and PC3-DR cell lines were sensitive to single-agent capivasertib and AZD8186. In fact, the PC3-DR cell line was especially sensitive, probably due to PTEN-loss-induced hyperactivation of the PI3K/AKT pathway and the resulting decrease in ERK phosphorylation. In contrast, the PTEN-wt DU145-DR cells were poorly sensitive to single-agent capivasertib or AZD8186. DU145-DR cells were less sensitive than DU145 cells, likely because the inhibition of the PI3K/AKT pathway may have been counteracted by the hyperactivation of the MEK/ERK pathway. In line with previous reports on MEK inhibition (Park et al., 2015), selumetinib treatment alone had a negligible effect on cell growth in both PC3-DR and DU145-DR cells. In contrast, selumetinib+AZD8186 had a synergistic effect in both DU145 and DU145-DR cells, with a greater synergism observed in docetaxel-resistant cells, probably due to the high dependence of these cells on the PI3K/AKT and MEK/ERK pathways. Intriguingly, we did not observe a reduction of S6 activation after selumetinib+AZD8186 either in the DU145-DR or the PC3-DR cells. As proposed by Xu and colleagues, this phenomenon could be due to a compensatory mechanism in which PI3Kβ/δ inhibition promotes PI3Kα-mediated mTOR activation, thereby inducing S6 phosphorylation (Xu et al., 2023).

The PI3K/AKT and MEK/ERK pathways negatively regulate each other’s activity: inhibition of the PI3K/AKT pathway results in upregulation of the MEK/ERK cascade (Lee et al., 2008; Wee et al., 2009; Park et al., 2015) and inhibition of the MEK/ERK pathway results in upregulation of the PI3K/AKT pathway (Hoeflich et al., 2009). In the present study, both capivasertib and AZD8186 promoted ERK1/2 phosphorylation in the PC3-DR cells, while selumetinib promoted PI3K/AKT hyperactivation in the DU145-DR cells. Previous studies have postulated that resistance to PI3K/AKT inhibitors could be due, at least in part, to the hyperactivation of the MEK/ERK pathway (Shorning et al., 2020), suggesting that the concomitant inhibition of both pathways would be necessary to decrease cell viability and increase apoptosis.

Our findings suggest that in view of the close crosstalk between the PI3K/AKT and MEK/ERK pathways, combination therapy with PI3K/AKT and MEK/ERK inhibitors could be a promising therapeutic strategy. In fact, several preclinical and clinical studies in solid tumors have assessed the impact of dual inhibition of the two pathways, but with conflicting results (Kinkade et al., 2008; Shimizu et al., 2012; Ewald et al., 2014; Bedard et al., 2015; Park et al., 2015; Toren et al., 2016; Marques et al., 2020). Although some of these studies were carried out in mCRPC models treated with hormonal therapies, to the best of our knowledge, no studies have analyzed the efficacy of such a combination in docetaxel-resistant mCRPC patients. Taking into account that docetaxel remains crucial in the current treatment landscape of either mCSPC and mCRPC patients, the development of new effective regimens in docetaxel-refractory setting remains an important challenge. Interestingly, our *in vivo* results suggest that the combination of selumetinib+AZD8186 might be more effective than cabazitaxel, which is currently considered one of the standard treatments for patients who progress after docetaxel therapy. Therefore, our study proposes a potential new and effective biomarker-selected therapeutic combination for docetaxel-resistant patients.

As we have shown, PTEN status plays a key role in treatment effectiveness. PTEN-knockdown DU145-DR cells were significantly more resistant to selumetinib+AZD8186 than PTEN-wt cells. Moreover, although several early-phase clinical studies of dual PI3K/AKT and MEK/ERK inhibition have reported high levels of toxicity (Shimizu et al., 2012; Bedard et al., 2015), we observed no toxicity in mice treated with selumetinib+AZD8186, suggesting that using an isoform-selective PI3K inhibitor like AZD8186 would allow us to optimize combination therapy and minimize toxicity.

## 5 Conclusion

Selumetinib+AZD8186 showed high antiproliferative and proapoptotic activity in PTEN-wt mCRPC cell lines *in vitro* and significantly decreased tumor growth in PTEN-wt docetaxel-resistant xenograft mouse models *in vivo*. Our findings suggest that selumetinib+AZD8186 could be a highly effective therapeutic strategy in patients with PTEN-wt docetaxel-resistant mCRPC, in whom, unlike PTEN-loss patients, a clinical benefit of treatment with single-agent PI3K and AKT inhibitors has not been demonstrated. A phase I-II trial is warranted to characterize the safety and efficacy of selumetinib+AZD8186 in PTEN-wt mCRPC patients.

## Data Availability

The original contributions presented in the study are included in the article/Supplementary Materials, further inquiries can be directed to the corresponding authors.
