# Supplementary material for: Dual inhibition of MEK and PI3Kβ/δ–a potential therapeutic strategy in PTEN-wild-type docetaxel-resistant metastatic prostate cancer
Source: Front Pharmacol. 2024 Jan 22;15:1331648. doi: 10.3389/fphar.2024.1331648 (PMC10838968; doi:10.3389/fphar.2024.1331648)
Supplement: Supplementary file 1 [file Table1.DOCX]

Supplementary Material

**Dual inhibition of MEK and PI3Kβ/δ – A potential therapeutic strategy in PTEN-wild-type docetaxel-resistant metastatic prostate cancer**

Vicenç Ruiz de Porras, Adrià Bernat-Peguera, Clara Alcon, Fernando Laguía, Maria Fernández-Saorín, Natalia Jimenez, Ana Senan-Salinas, Carme Solé-Blanch, Andrea Feu, Mercedes Marin-Aguilera, Juan Carlos Pardo, Maria Ochoa-de-Olza, Joan Montero, Begoña Mellado, Albert Font

# Supplementary Figures and Tables

## Supplementary Figures

**Supplementary Fig. 1**

**
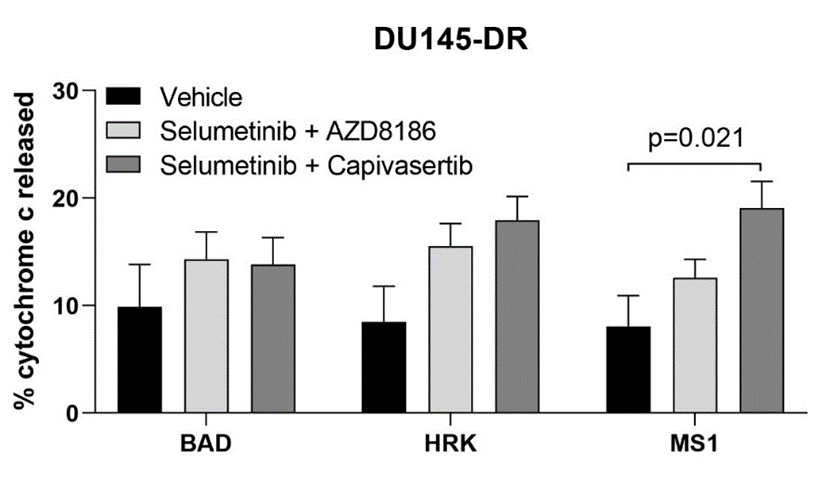
**

**Supplementary Fig. 1 – BCL-xL and MCL-1 resistance in PTEN-wild-type DU145-DR cells after treatment with selumetinib+capivasertib or selumetinib+AZD8186.**

The contribution of each antiapoptotic protein (BCL-2/BCL-xL dependence BAD peptide; BCL-xL dependence HRK peptide; and MCL-1 dependence MS1 peptide) to acquired resistance to selumetinib+AZD8186 or selumetinib+capivasertib in PTEN-wild-type DU145-DR cells. Results represent the difference in % cytochrome c released (mean ± SEM) between treated vs non-treated cells after exposure to the peptides. Results were obtained from at least three independent biological replicates. P-values were calculated using a two-way ANOVA test for multiple comparisons. Significance was set at p≤0.05.

**1.2. Supplementary Tables**

**Supplementary Table 1 –** Docetaxel IC50 values for each cell line used in this study (32)

| **Cell line** | **DU145** | **DU145-DR** | **PC3** | **PC3-DR** |
| --- | --- | --- | --- | --- |
| **IC50 value (95% CI) nM** | 6.15 (5.32-7.11) | 12.35 (11.14-13.70) | 15.44 (11.44-20.84) | 30.89 (22.37-42.66) |

**Supplementary Table 2 –** Growth and molecular characteristics of the prostate cancer cell lines used in the study.

| **Cell line** | **Hormone response** | **AR** | **PTEN** | **PI3Kα** | **PI3Kβ** | **PI3Kδ** |
| --- | --- | --- | --- | --- | --- | --- |
| **DU145** | CRPC | Neg | WT | WT | WT | WT |
| **PC3** | CRPC | Neg | Loss | WT | WT | WT |

**Supplementary Table 3 –** Antibodies used in the study

| **Primary Antibodies** | | | | |
| --- | --- | --- | --- | --- |
| **Antigen** | **Company** | **Catalog #** | **Type** | **Use and dilution**  **(western blot)** |
| AKT | Cell Signaling | 4691S | Rabbit mAb | 1:1000 |
| Phospho-AKT (S473) | Cell Signaling | 4060S | Rabbit mAb | 1:1000 |
| ERK1/2 (p44/42) | Cell Signaling | 9102S | Rabbit mAb | 1:1000 |
| Phospho-ERK1/2 (p44/42) (T202/Y204) | Cell Signaling | 4370S | Rabbit mAb | 1:1000 |
| PTEN | Cell Signaling | 9188T | Rabbit mAb | 1:1000 |
| Phospho-S6 Ribosomal protein (S235/236) | Cell Signaling | 4858T | Rabbit mAb | 1:1000 |
| S6 Ribosomal protein | Cell Signaling | 2217S | Rabbit mAb | 1:1000 |
| Phospho-p90RSK (S380) | Cell Signaling | 11989T | Rabbit mAb | 1:1000 |
| RSK1/RSK2/RSK3 | Cell Signaling | 9355T | Rabbit mAb | 1:1000 |
| Phospho-GSK3β (S9) | Cell Signaling | 5558T | Rabbit mAb | 1:1000 |
| GSK3β | Cell Signaling | 9315S | Rabbit mAb | 1:1000 |
| Cyclin D1 | Abcam | ab134175 | Rabbit mAb | 1:1000 |
| CDK4 | Cell Signaling | 12790 | Rabbit mAb | 1:1000 |
| CDK6 | Cell Signaling | 3136 | Mouse mAb | 1:1000 |
| BCL-2 | Abcam |  | Rabbit mAb | 1:1000 |
| α-Tubulin | Sigma | T6074 | Mouse mAb | 1:10000 |
| β-actin | Sigma | A2066 | Rabbit mAb | 1:2000 |
| GAPDH | Sigma | G9545 | Rabbit mAb | 1:1000 |
| **Secondary antibodies** | | | | |
| IRDye anti-rabbit | LICOR  Biosciences | 926-68071 |  | 1:10000 |
| IRDye anti-mouse | LICOR  Biosciences | 926-32210 |  | 1:10000 |
